# Bax Inhibitor-1 down-regulation in the progression of chronic liver diseases

**DOI:** 10.1186/1471-230X-10-35

**Published:** 2010-04-01

**Authors:** Andromachi Kotsafti, Fabio Farinati, Romilda Cardin, Patrizia Burra, Marina Bortolami

**Affiliations:** 1 Department of Surgical and Gastroenterological Sciences, University of Padua, Padua, Italy

## Abstract

**Background:**

Bax inhibitor-1 (BI-1) is an evolutionary conserved endoplasmic reticulum protein that, when overexpressed in mammalian cells, suppresses the apoptosis induced by Bax, a pro-apoptotic member of the Bcl-2 family. The aims of this study were: (1) to clarify the role of intrinsic anti- and pro-apoptotic mediators, evaluating Bax and BI-1 mRNA and protein expressions in liver tissues from patients with different degrees of liver damage; (2) to determine whether HCV and HBV infections modulate said expression.

**Methods:**

We examined 62 patients: 39 with chronic hepatitis (CH) (31 HCV-related and 8 HBV-related); 7 with cirrhosis (6 HCV-related and 1 HBV-related); 13 with hepatocellular carcinoma (HCC) [7 in viral cirrhosis (6 HCV- and 1 HBV-related), 6 in non-viral cirrhosis]; and 3 controls. Bax and BI-1 mRNAs were quantified by real-time PCR, and BI-1 protein expression by Western blot.

**Results:**

CH tissues expressed significantly higher BI-1 mRNA levels than cirrhotic tissues surrounding HCC (P < 0.0001) or HCC (P < 0.0001). Significantly higher Bax transcripts were observed in HCV-genotype-1-related than in HCV-genotype-3-related CH (P = 0.033). A positive correlation emerged between BI-1 and Bax transcripts in CH tissues, even when HCV-related CH and HCV-genotype-1-related CH were considered alone (P = 0.0007, P = 0.0005 and P = 0.0017, respectively).

**Conclusions:**

BI-1 expression is down-regulated as liver damage progresses. The high BI-1 mRNAs levels observed in early liver disease may protect virus-infected cells against apoptosis, while their progressive downregulation may facilitate hepatocellular carcinogenesis. HCV genotype seems to have a relevant role in Bax transcript expression.

## Background

Hepatocellular carcinoma (HCC) is a late complication of chronic viral hepatitis and derives both from the chronic inflammatory process, with consequent free radical over-expression [1], cytokines [2] and growth factors release [3], and from the capacity of viral proteins to modulate the proliferation and death of infected hepatocytes. It has been indeed suggested that dysregulation of the apoptotic process in infected cells might be crucial in favoring the chronicity of infection, the failure of antiviral treatment and neoplastic transformation [4]. Much attention has recently been focused on the intrinsic pathways of the apoptotic process, and in particular on Bax and Bax inhibitor 1 (BI-1) [5,6].

Bax is a pro-apoptotic protein, a member of the Bcl-2 family that includes pro- and anti-apoptotic proteins [7,8]. Bax-induced apoptosis is associated with a change in Bax cellular localization, reflecting its ability to undergo a change from a soluble to an integral membrane conformation [9]. Indeed, Bax is usually found in the cytosol but, when activated, it moves to the mitochondria and induces cytochrome c release [10,11].

In the cytosol, Bax encounters different environmental conditions (e.g. pH, ion concentrations, ATP, NADH, oxygen and many other parameters) that may induce changes in Bax structure, with loss of ability to access and interact with other proteins. It has been shown that some anti-apoptotic proteins may hijack Bax in the cytosol, thus preventing Bax oligomerization in the mitochondrial outer membrane [12], which seems to be an important step in the activation of Bax, making the mitochondrial membranes permeable and triggering the executive phase of the apoptotic process (with caspase 3 and caspase 9 activation) [13].

Other proteins may bind the active form of Bax in the mitochondria, however, thereby preventing Bax oligomerization and the subsequent cytochrome *c *release (clusterin, Bcl-2). One such protein is Bcl-2, which may bind Bax and form heterodimers, consequently inhibiting Bax activity [14].

Human Bax inhibitor-1 has also been identified as a suppressor of Bax-mediated cell death in yeast [15]. BI-1 is an anti-apoptotic integral membrane protein, located mainly in the intracellular membranes of endoplasmic reticulum (ER) [16]. Its over-expression in mammalian cells suppresses apoptosis induced by Bax, staurosporine and growth factor deprivation. BI-1 is presumably not needed for the physiological regulation of developmental programmed cell death, since the liver tissues of BI-1-deficient mice are histologically normal [17]. The anti-apoptotic mechanism of BI-1 involves the suppression of Bax activation and translocation to mitochondria, preserving the mitochondria membrane potential and mitochondrial morphology, and preventing the activation of post-mitochondrial caspases. BI-1 function is also associated with the regulation of intracellular Ca^2+ ^omeostasis [18].

The anti-apoptotic efficiency of BI-1 is also confirmed by the fact that BI-1 antisense oligonucleotide can promote apoptosis in some tumor lines [15]. Little is known as yet about the BI-1 protein's functions in human liver diseases.

Given the importance of the biological activities of Bax and BI-1, the aims of this study were to establish whether Bax and BI-1 are expressed differently in the various stages of chronic liver damage, from chronic hepatitis to HCC.

## Methods

### Patients

This study involved 62 patients.

A *first group *included 39 patients with chronic hepatitis (CH): 31 HCV-related (10 females, 21 males; mean age 40.2 ± 12.2 years; range 20-67; HCV-genotypes: 18 HCV-1, 6 HCV-2, 4 HCV-3, 3 HCV-4), and 8 HBV-related (1 female, 7 males; mean age 39.5 ± 10.7 years; range 28-56).

The *second group *included 7 cirrhotic patients (CIRR): 1 HBV-related (a 56-year-old female) and 6 HCV-related (1 female, 5 males; mean age 44 ± 14.4 years; range 33-69).

The *third group *consisted of 13 patients with HCC in cirrhosis: 6 HCV-related (2 females, 4 males; mean age 56.8 ± 16.9 years; range 23-69) and 1 HBV-related (a 55-year-old male); and 6 patients with HCC in non-virus-related cirrhosis (2 females, 4 males; mean age 61.5 ± 8.5 years; range 46-72). As "control" specimens (Control), liver biopsy samples from 3 patients who had undergone cholecystectomy were considered (2 females, 1 male; mean age 44.6 ± 18.6 years; range 25-62).

The diagnosis of chronic HCV infection was obtained on the basis of HCV-RNA positivity by polymerase chain reaction (PCR), persistently abnormal transaminases for at least 12 months, and a compatible histology. Histological diagnoses were made by a single pathologist and scored, according to Ishak's classification [19], in all biopsy samples. Before biopsy, each patient was tested to measure HCV antibodies.

All the following studies were performed prior to any treatment.

All patients, Caucasian Mediterranean, enrolled in this study, were recruited from the Department of Surgical and Gastroenterological Sciences, Division of Gastroenterology, School of Medicine, University of Padua, Italy.

Informed consent was obtained from all patients and the study protocol was approved by our Institute's Ethics Committee.

### Liver samples

During a US-guided procedure (with a 16-18 gauge modified Menghini needle), liver biopsies were obtained from patients with chronic hepatitis or liver cirrhosis according to a standard protocol. One part of the liver specimen, at least 2 cm long, was used for diagnostic purposes while the remainder was immediately frozen in liquid nitrogen and stored at -80°C.

Tissue samples were obtained from HCC at the time of surgical resection. Tumor tissues used for mRNA extraction were macroscopically selected in the middle of the nodule. The corresponding non-cancerous tissues (PHCC) were taken at least 1 cm (more where possible) from the edge of the tumor in the resected specimen. To check for any presence of infiltrating tumor cells, a slice of the sample was fixed in buffered formaldehyde, stained with hematoxylin and eosin and examined by the pathologist. In all cases, care was taken during the surgical procedure to avoid contamination between cancerous and non-cancerous samples.

All surgical liver specimens were cut into small pieces, immediately snap frozen in liquid nitrogen, then stored at -80°C.

The routine histological analysis was done in blind fashion by the pathologist. HCC lesions were classified as well-, moderately- or poorly-differentiated according to the Edmonson & Steiner criteria [20], grading the specimens on the basis of the predominant findings.

### Virological and biochemical assessments

In all HCV-infected patients, HCV-specific serum antibodies were detected by enzyme immunoassay (EIA-II; Ortho Diagnostic System) and confirmed by recombinant immunoblot assay (RIBA-II; Ortho Diagnostic System) according to the manufacturer's instructions.

HCV RNA was detected with a standardized polymerase chain reaction (PCR) (Amplicor, Roche Diagnostic Systems, Neuilly, France) in the total RNA extracted from biopsies.

HCV genotype was determined by the Inno-Lipa II HCV method (Innogenetics S.A., Gent Belgium). HCV genotypes were classified as genotype 1, subtypes 1a and 1b, and the remaining subtypes of types 2, 3, and 4 were pooled under each corresponding genotype.

HBV serum markers were tested by radio-immune assay (RIA) (Abbott, Chicago-Illinois, USA), while HBV-DNA was tested with a commercially-available fluid phase hybridization assay (Abbott, Chicago-Illinois, USA).

Data regarding transaminases were obtained.

### RNA isolation

Total RNA was extracted from frozen hepatic tissue with acid guanidium thiocyanate-phenol-chloroform according to the Chomczynski and Sacchi method [21]. RNA concentrations were quantified spectrophotometrically. Integrity of the RNA sample was assessed by electrophoresis on a 2% agarose gel (FMC Bio Product, Rockland, ME, USA) containing ethidium bromide. The quality of the RNA isolated was also assessed using the RNA 6000 Nano Assay and the Agilent 2100 bioanalyzer (Agilent Technologies, Palo Alto, CA, USA).

### Reverse transcription

For the synthesis of complementary DNA (cDNA), 2 μg of RNA were reverse transcribed in a final volume of 40 μl in the presence of 1X PCR buffer, 1 mM each of dNTPs (dATP, dTTP, dCTP, dGTP), 1 U RNase inhibitor, 2.5 μM random hexamers, and 2.5 U of murine leukemia virus (Perkin Elmer, Foster City, CA, USA).

The reverse transcription reaction was completed at 25°C for 10 min, 42°C for 15 min and 99°C for 5 min, in a Perkin Elmer GeneAmp PCR System 2400.

The cDNA was stored at -20°C.

### Primers

Oligonucleotide primers were designed with the Primer Express software rel. 1.0 (ABI/PE Applied Biosystems, Foster City, CA, USA) and synthesized by Primm (San Raffaele, Milano-Italy). Nucleotide sequences for the sense and antisense primers used for real-time PCR were: 5'-CTTTTGCTTCAGGGTTTCATCC-3', 5'-TTGAGACACTC GCTCAGCTTCT-3' for Bax [ENST00000356483], and the length of this amplicon was 119 bp; 5'-TCTATGCAAGTTTTGCCCTTTGTA-3', 5'-GCCAGCCTGAATGAAATGA-3' for BI-1 [ENST00000267115], and the length of this amplicon was 84 bp; 5'-CCTGGCACCCAGCACAA-3', 5'-GCCGATCCACACGGAGTACT for β-actin [ENST00000158302], and the length of this amplicon was 70 bp.

### PCR product analysis

PCR products underwent vertical electrophoresis on a 0.75 mm thick, non-denaturing 6% acrylamide/bis-acrylamide gel with 5% glycerol. The silver-nitrate-stained bands were scanned on a densitometer and image analyzer system (Quantity-one- BIO-RAD Hercules, CA, USA).

### DNA purification

Purified DNAs were obtained using the MinElute PCR Purification Kit according to the manufacturer's protocol. The concentration of the purified amplicons was quantified spectrophotometrically with the Biophotometer 6131 (Eppendorf, Hamburg, Germany) and the PicoGreen ds-DNA quantitation reagent and kits.

Fluorescence was measured with the LS-5 Luminescence Spectrometer (Perkin Elmer, Foster City, CA) using 480 nm excitation and 520 nm emission.

### Quantitative absolute real-time PCR

Real-time PCR was conducted in an ABI 7900 Sequence Detection System (Applied Biosystems, Foster City, CA, USA) using SYBR Green I [22]. The reaction was obtained on 96-well plates, in a 25 μL final volume containing 1X TaqMan buffer, 5.5 mmol of MgCl_2_, 200 μmol of nucleotides with dUTP, 0.25 U of AmpliTaq Gold Polymerase (SYBR Green Master Mix), 300 nM of each primer and 200 ng of cDNA template. After one 2-min step at 50°C and a second step at 95°C for 10 min, samples underwent 45 cycles of 45 s at 94°C and then: 45 s at 60°C for Bax and β-actin; 45 s at 62°C for BI-1. A final extension step was performed at 60°C for 10 min. All tests were performed in triplicate. Samples in which the cDNA was omitted were used as negative controls. All the technical details of this method are given in Table 1.

**Table 1:** Characteristic values relating to the expression of each gene considered by Real time PCR

*Gene*	*Threshold*	*Slope*	*Efficiency* *E = (10*^*-1/slope*^*)-1*	*R*^2^	*Range* *(copies/μl)*
**β-*actin ****(n.24)*	***0.078***	***-3.34 ± 0.03***	***98.37 ± 3.43***	***0.998***	***10*^8^*-10*^2^**
***Bax ****(n.15)*	***0.104***	***-3.51 ± 0.05***	***94.67 ± 1.99***	***0.998***	***10*^8^*-10*^3^**
***BI-1 ****(n.18)*	***0.144***	***-3.48 ± 0.03***	***93.48 ± 1.34***	***0.999***	***10*^8^*-10*^2^**

Threshold value, manually adjusted, was maintained for all the standard curve analyzed. Table report the mean ±SD of the slope and efficiency value relative to n. 24, 15 and 18 standard curves of β-actin, Bax and BI-1 respectivelly. Efficiency was calculated from the slope using the equation E = (10^-1/slope^) -1. The correlation coefficient (square values R^2^) and range of standard curves are also reported.

### Gene expression quantification

The amounts of mRNA in the unknown samples were determined from the standard curves of the gene of interest [23]. Standards curves were generated using serial diluition (1:10) from 10^8 ^to 10^2 ^copies/μl of a reference sample.

Data were expressed as the ratio of the transcript amounts of the gene of interest to the β-actin transcript used as housekeeping gene.

### Western blot analysis

Total protein extracts were obtained by homogenizing liver tissues with RIPA lysis buffer (20 mM TrisHCl pH 7.4, 150 mM NaCl, 5 mM ethylene diamine tetra-acetic acid [EDTA], 1.5% Niaproof, 1 mM sodium orthovanadate Na_3_VO_4 _0.1% sodium dodecyl sulfate [SDS]). Protein concentration was determined using the RC DC Protein Assay (Bio-Rad, Hercules, CA, USA). 40 μg of boiled proteins were loaded onto the gel. Proteins were separated by SDS-PAGE, transferred onto nitrocellulose membrane (Hybond ECL, GE Healthcare, Buckinghamshire, UK) and blocked with fat-free milk [5% in Tween-phosphate-buffered saline (PBS)] for 1 h. Membranes were probed with the primary mouse monoclonal antibody against human BI-1 (1:200) and the primary mouse monoclonal antibody against β-actin (1:1000) (Santa Cruz Biotechnologies, California, USA). After incubation with the secondary antibody (goat anti-mouse IgG, at a dilution of 1:10000), immunoreactive proteins were visualized by chemiluminescence using SuperSignal WestPico Chemiluminescent Substrate (Pierce, Rockford, Illinois, USA) and captured on X-ray film (Hyperfilm™ ECL, GE Healthcare, Buckinghamshire, UK). Molecular sizing was carried out using the Full-Range Rainbow Molecular weight Marker (GE Healthcare, Buckinghamshire, UK). Exposed films were digitized and the bands were semi-quantitatively evaluated by densitometric analysis. BI-1 protein expression levels were thus normalized to those of the housekeeping gene, β-actin.

### Statistical analysis

All results are given as mean values ± SD (standard deviation). Statistical analyses were performed using the StatSoft software, rel. 5.0 (Tulsa, OK, USA). Differences between groups were analyzed with the Kruskal-Wallis test or Mann-Whitney U test, as appropriate. Two-tailed P value < 0.05 were deemed to be significant. The relationship between two variables was determined using Pearson's correlation analysis, accepting a P < 0.05 as significant.

## Results

**Figure 1:**
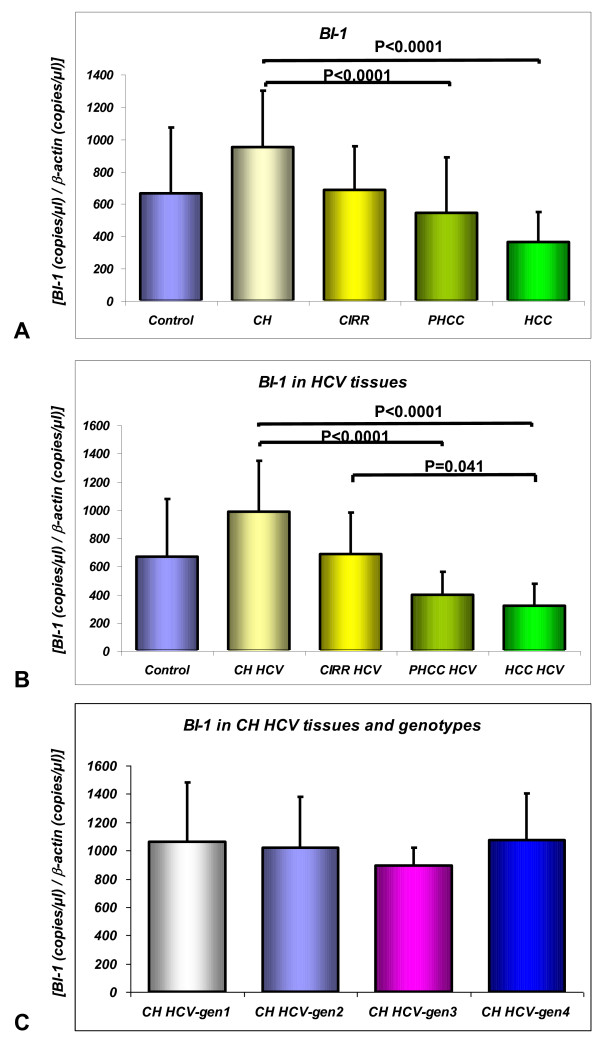
**BI-1 mRNA analysis by quantitative absolute real-time PCR using SYBR Green I: in control specimens (Control), liver samples from patients with chronic hepatitis (CH), cirrhosis (CIRR), cirrhotic tissues surrounding hepatocellular carcinoma (PHCC) and HCC (1A); in HCV-infected liver tissues (1B); in CH HCV-related tissues according to genotypes (1C)**. The unknown BI-1 and β-actin mRNA amounts in the samples were extrapolated by the respective standard curves performed using serial diluition (1:10) from 10^8 ^to 10^2 ^copies/μl of a reference sample. Form each sample the normalized amount of the BI-1 transcripts was obtained by the ratio of BI-1 to β-actin, used as housekeeping gene. Data are expressed as mean values ± SD.

**Figure 2:**
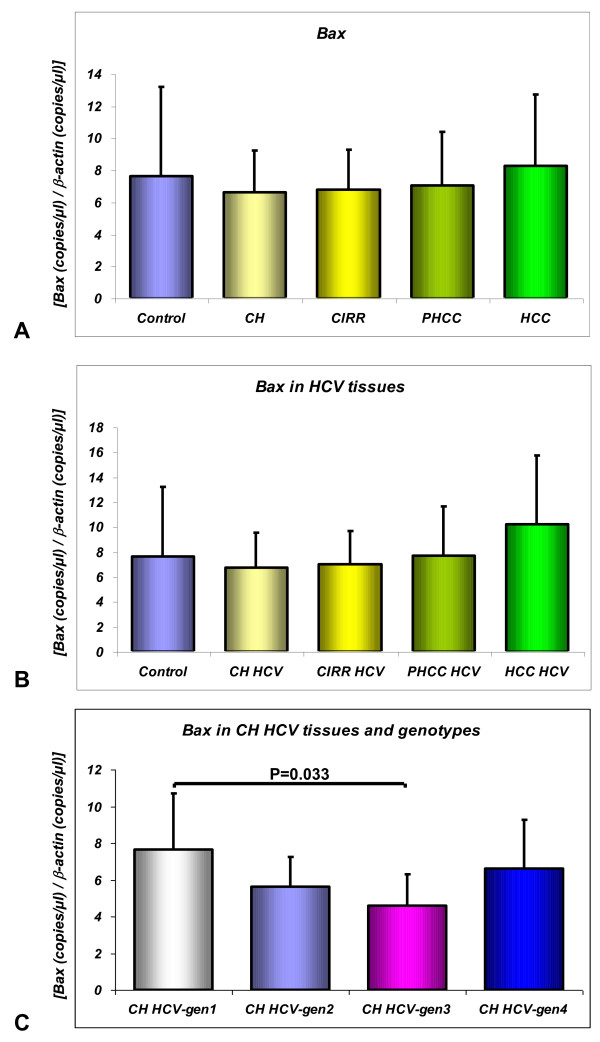
**Bax mRNA analysis by quantitative absolute real-time PCR using SYBR Green I: in control specimens (Control), liver samples from patients with chronic hepatitis (CH), cirrhosis (CIRR), cirrhotic tissues surrounding hepatocellular carcinoma (PHCC) and HCC (2A); in HCV-infected liver tissues (2B); in CH HCV-related tissues according to genotypes (2C)**. The unknown Bax and β-actin mRNA amounts in the samples were extrapolated by the respective standard curves performed using serial diluition (1:10) from 10^8 ^to 10^2 ^copies/μl of a reference sample. Form each sample the normalized amount of the BI-1transcripts was obtained by the ratio of Bax to β-actin, used as housekeeping gene. Data are expressed as mean values ± SD.

The BI-1 and Bax mRNA levels in the livers are shown in Figure 1 and Figure 2 respectively.

### BI-1 mRNA expression

As concerns BI-1 expression, a statistically significant difference (P < 0.0001 by Kruskal Wallis test) emerged between the five groups considered (Control, CH, CIRR, PHCC, HCC). CH tissues expressed the highest levels of BI-1 mRNA (950.81 ± 348.6), significant higher than in PHCC (543.1 ± 345.4; P < 0.0001) or HCC (365.92 ± 185.52; P < 0.0001) tissues (Figure 1A). CIRR expressed the same amount of BI-1, whether it was associated with HCC or not. There was no difference in BI-1 mRNA levels between tissues with HCV versus HBV-related CH (984.83 ± 365.33 vs 819.00 ± 250.72).

Statistically significant differences in BI-1 expression were observed between CH HCV and PHCC HCV (p < 0.0001) and HCC HCV (p < 0.001) and between CIRR HCV and HCC HCV (p = 0.041) (Figure 1B).

No statistically significant difference in BI-1 expression was found in CH HCV tissues according to the genotype (Figure 1C).

No difference between virus- and non virus-related HCC was detected (data non shown).

### Bax mRNA expression

Bax transcript levels did not differ significantly between Control tissues (7.623 ± 5.5) and all the pathological tissues considered (CH 6.618 ± 2.6; CIRR 6.808 ± 2.4; PHCC 7.029 ± 3.3; HCC 8.294 ± 4.4) (Figure 2A). Nor were there any differences in CH according to its etiology (CH HCV 6.749 ± 2.8; CH HBV 6.111 ± 1.6). No differences in Bax mRNA expression were found considering only HCV-infected tissues (Figure 2B). When only cases of CH with HCV-related hepatitis were considered, according to the genotype involved, patients with HCV-genotype-1 infection showed a statistically higher Bax mRNA expression than in patients with HCV-genotype-3 infection (7.620 ± 3.0 vs 4.605 ± 1.70, P = 0.033) (Figure 2C).

No difference between virus- and non virus-related HCC was detected (data non shown).

### Western blot

**Figure 3:**
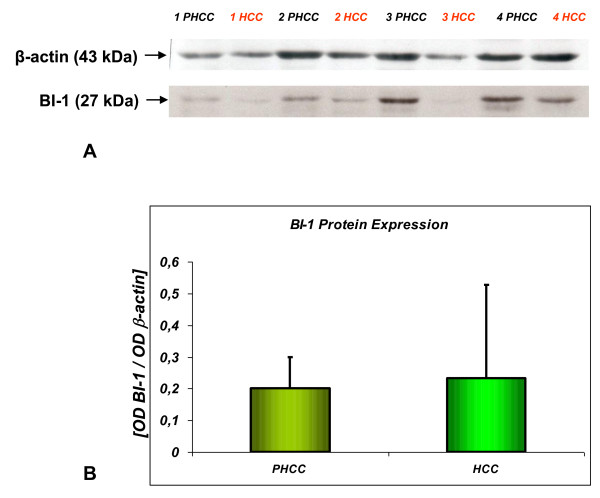
**BI-1 protein expression in HCC and tissues surrounding HCC**. **(3A):** Western blot analysis of BI-1 protein expression in cirrhotic liver tissues surrounding hepatocellular carcinoma (PHCC) and in HCC tissues obtained from the same patient. Proteins were loaded onto 15% SDS-polyacrylamide gels and transferred to nitrocellulose membranes. Blots were incubated with anti-BI-1 and anti β-actin antibodies, followed by detection using an ECL method. **(3B)**: Results of the densitometric analysis of BI-1. Data are expressed as the optical density ratio of BI-1 to β-actin. Values are reported as mean values ± SD.

BI-1 protein expression was only evaluated in surgical liver tissues from 15 HCC patients, both in the cancer and in the surrounding cirrhotic tissue. Like the BI-1 mRNA transcripts, BI-1 protein expression did not differ in these tissues (Figure 3).

### Correlations

**Figure 4:**
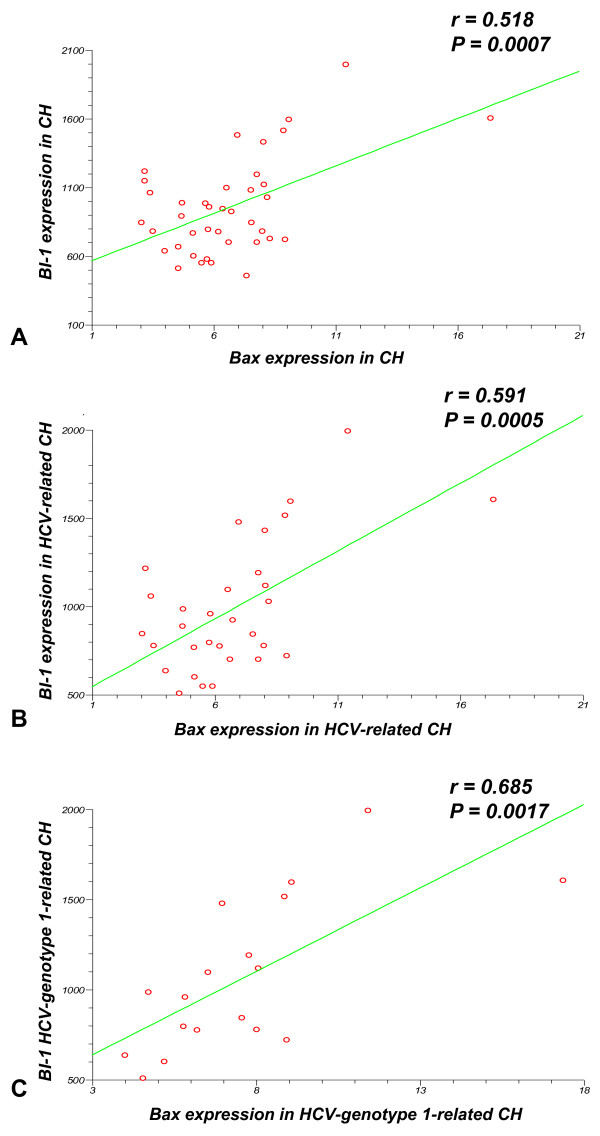
**Results of the linear regression analysis in tissues from patients with CH**. (**4A**): Linear regression analysis of BI-1/β-actin vs Bax/β-actin in all CH tissues; (**4B**): in HCV infected CH tissues; (**4C**): in HCV-genotype-1-related CH.

A significant positive correlation emerged between BI-1 and Bax mRNA expression in CH tissues (r = 0.51; P = 0.0007) (Figure 4A). The correlations between BI-1 and Bax mRNA expression were also significant when patients with HCV-related CH (r = 0.59; P = 0.0005) (Figure 4B) or HCV-genotype-1-related CH (r = 0.68; P = 0.0017) (Figure 4C) were considered separately.

No correlation in CH with disease "activity" (AST/ALT levels) was detected.

## Discussion

This study sought possible differences in the expression of the anti-apoptotic BI-1 and pro-apoptotic Bax factors in liver tissues from patients with different severity of liver damage, from chronic viral hepatitis to cirrhosis and finally HCC, with a view to examining its biological role in the progression of liver disease towards cancer, naturally history in which little is known about the expression of BI-1 and Bax.

Bax mRNA transcripts were much the same in the normal, cirrhotic, and HCC tissues analyzed, suggesting that the presence of a constitutive expression of Bax in the liver is not significantly up- or down-regulated during the progression of liver diseases. Bax has not always been found located in the cytosol, however, and a constitutive mitochondrial location of Bax in healthy cells has been described both in cell culture [24] and in experimental animals, in different organs [16,17]. Moreover, the activation of Bax during apoptosis does not usually require an increase in this gene transcription. These findings are consistent with our results and suggest that Bax mRNA levels are not differently modulated in normal (even though the number of "control" subjects in our series is limited) and diseased livers, so they do not change with the progression of chronic liver damage. Bax transcripts however, seems to be modulated by the HCV genotype, at least judging from the greater increase of Bax in HCV-genotype-1-related CH as compared with HCV-genotype-3-related CH.

Activation and functioning of Bax is a complex process. Many recent studies have described the conformation of native cytosolic Bax and how the Bax protein can interact with other proteins undergoing conformational modifications (such as the switch from a cytosolic to a mitochondrial, membrane-insert protein) during apoptosis [25].

Inside the cell, Bax is subject to the effect of many environmental factors, which vary in time, slightly loosening the globular structure and allowing protein-protein interaction. Under physiological conditions, this loosening of the structure may be an early event that is necessary but not sufficient to induce a transition in Bax conformation. The binding of additional membrane compounds or other BH3-containing proteins might be the determining event that triggers the conformational change [12,26].

Bax is actually a multidomain protein containing the BH-1, BH-2 and BH-3 domains, so it may form complexes such as: Bax/Bax, Bax/Bcl-2, Bax/BclX_L _and thus drive a different (pro or anti) intracellular apoptotic pathway activation [27]. A reduced Bax expression has been described in many human diseases, primarily tumors, such as in ovarian [28] and cervical cancer [29] a fact that, at least in our experience is not confirmed in HCC.

Conversely, we found that BI-1 mRNA transcripts dropped significantly with the progression of liver damage from CH to cirrhosis and HCC, suggesting a crucial role for BI-1 in regulating the apoptotic pathways, particularly in early liver disease. The drop in BI-1 involved similarly both HCC and the cirrhotic tissues surrounding the tumor, thus indicating the presence of a "field defect" that involves also virus- and non virus-related HCC. BI-1 is an integral membrane protein localized in ER membranes and protects cells from apoptosis induced by ER stress. Acute and chronic liver diseases are characterized by inflammatory processes with enhanced expression of various pro- and anti-inflammatory cytokines, toxic metabolites, ROS (reactive oxygen species) and bile acids. Therefore the increase of BI-1 mRNA expression may represent a protective effect with respect to the virus-induced inflammatory response.

The mechanism by which BI-1 regulates ER Ca^++ ^fluxes remains unclear. Recently it was demonstrated that the effect of BI-1 on ER Ca^++ ^permeability is pH-dependent. Acidic condition promotes BI-1 oligomerization inducing the creation of Ca^++ ^channel and consequent Ca^++ ^release from ER [30].

BI-1 is also a critical regulator of ER stress response in the development of obesity-associated insulin resistance, as reported by Bailly-Maitre B et al. [31]. The study of BI-1 expression in NAFLD (Non-Alcoholic Fatty Liver Disease) could be important to understand its molecular function also in metabolic disorders.

In liver cancer inflammatory cells colonize the liver and we also demonstrated, in a previous study, increased proinflammatory cytokines mRNA levels and lymphocytes and monocytes/macrophages density in tissues surrounding primary and secondary liver tumors [32]. These cells have an important role in inducing a pH reduction. Therefore the low BI-1 mRNAs expression found in HCC seems be also related to the decrease of inflammatory response and consequently to the local alkalinization.

It is well known that liver cell proliferation increases during chronic hepatitis while an impaired hepatocyte proliferation at the cirrhosis stage leads to a further selection of genetically altered, hyper-proliferating, pre-malignant clone of transformed hepatocytes and then tumor formation. In this scenario apoptosis is, in HCV-related hepatitis, relatively down-regulated with respect to the increased cell proliferation [33]. Also, in an our previous study, TUNEL analysis revealed a decrease of the number of apoptotic cells in HCC [34]. Therefore the low BI-1 mRNA expression showed in our HCC and peritumoral tissues in this study, with no difference between the two as confirmed by western blotting, support previous findings and may contribute to the carcinogenetic process through the inhibition of the apoptotic process, favoring tumor progression.

The statistically significant differences found in HCV tissues points to an involvement of viral proteins, and particularly the proteins of the C virus genotype 1, in modulating the apoptotic pathway. HCV has developed a range of mechanisms for evading the cell's defenses, modulating the apoptosis of infected cells as, for instance, the interaction with PKR (a dsRNA-dependent protein kinase).

NS5A (non-structural 5A) and E2 (envelope) HCV proteins may bind to and inactivate PKR [35,36]. This seems more evident in genotype 1 variant by the greatest homology found between E2 and PKR compared to the other genotypes.

The higher Bax levels found in HCV genotype 1 may be therefore indicative of an additional signaling pathway independent of PKR; for instance, the association of NS5A with the ER and Golgi [37] may represent an alternative pathway involved in Bax activation.

## Conclusion

The BI-1 expression decrease correlates closely with the progression of liver disease from CH to cirrhosis and then to HCC in HCV-infected tissues, suggesting a clear role of viral proteins in the induction of relevant event for liver damage. Therefore this down-regulation of BI-1 observed both in HCC and in the cirrhotic tissue surrounding the tumor, may well contribute to hepatocellular carcinogenesis and tumor progression. The lack of a difference between BI-1 expression in virus- versus non virus-related HCC points to a "field defect" not related to disease etiology. A deeper understanding of BI-1 and Bax expression, modulation and interaction with other factors involved in the apoptotic pathways could prove useful for the development of new approaches to the prevention and the treatment of liver cancer.

## List of Abbreviations

Bax: Bcl-2 associated X protein; BI-1: Bax Inhibitor-1; CH: Chronic Hepatitis; PHCC: Cirrhotic tissues surrounding Hepatocellular carcinoma; HCC: Hepatocellular carcinoma; HBV: Hepatitis B virus; HCV: Hepatitis C virus.

## Competing interests

The authors declare that they have no competing interests.

## Authors' contributions

AK performed the majority of experiments; FF involved in critical reading and helpful discussion of the manuscript; RC provided the collection of human liver biopsies; PB provided part of financial support of this work; MB designed the study and wrote the manuscript. All authors read and approved the final manuscript.

## Pre-publication history

The pre-publication history for this paper can be accessed here:

http://www.biomedcentral.com/1471-230X/10/35/prepub
